# Supplementary material for: A unified framework for herbivore-to-producer biomass ratio reveals the relative influence of four ecological factors
Source: Commun Biol. 2021 Jan 8;4:49. doi: 10.1038/s42003-020-01587-9 (PMC7794211; doi:10.1038/s42003-020-01587-9)
Supplement: Supplementary file 2 — Supplementary Information [file 42003_2020_1587_MOESM2_ESM.pdf]

Supplementary information

A unified framework for herbivore-to-producer biomass ratio reveals the relative influence of four ecological factors

Takehiro Kazama, Jotaro Urabe*, Masato Yamamichi, Kotaro Tokita, Xuwang Yin, Izumi Katano, Hideyuki Doi, Takehito Yoshida and Nelson G. Hairston Jr.

* author for correspondence: urabe@tohoku.ac.jp

Supplementary Figure 1
Supplementary Figure 2
Supplementary Figure 3
Supplementary Figure 4
Supplementary Figure 5
Supplementary Figure 6
Supplementary Figure 7
Supplementary Figure 8
Supplementary Figure 9
Supplementary Table 1
Supplementary Table 2
Supplementary Table 3

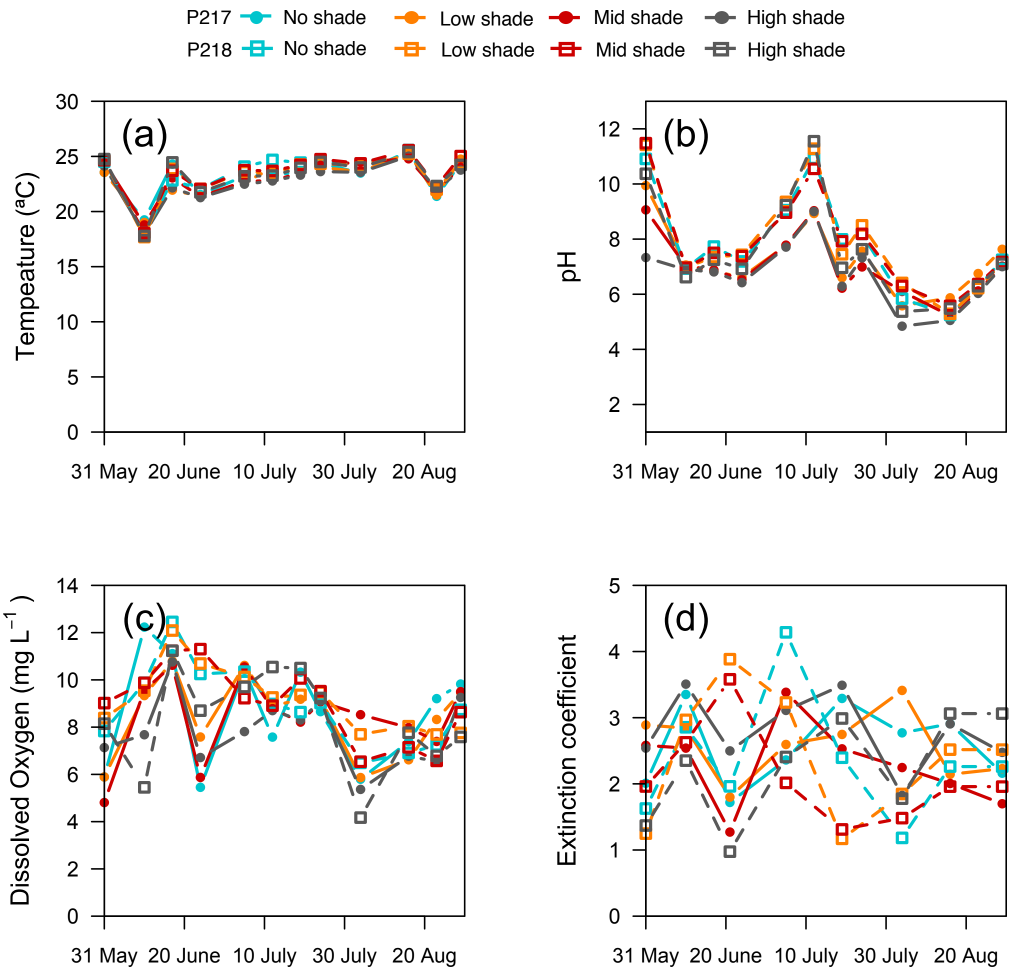

Supplementary Figure 1. Temporal changes in mean water temperature (a), pH (b), and dissolved oxygen (c) in the water column, and extinction coefficient (m^{-1}) (d) in the no-shade (blue), low-shade (orange), mid-shade (red), and high-shade sections (gray) of pond 217 (circle and sold line) and pond 218 (square and dashed line).

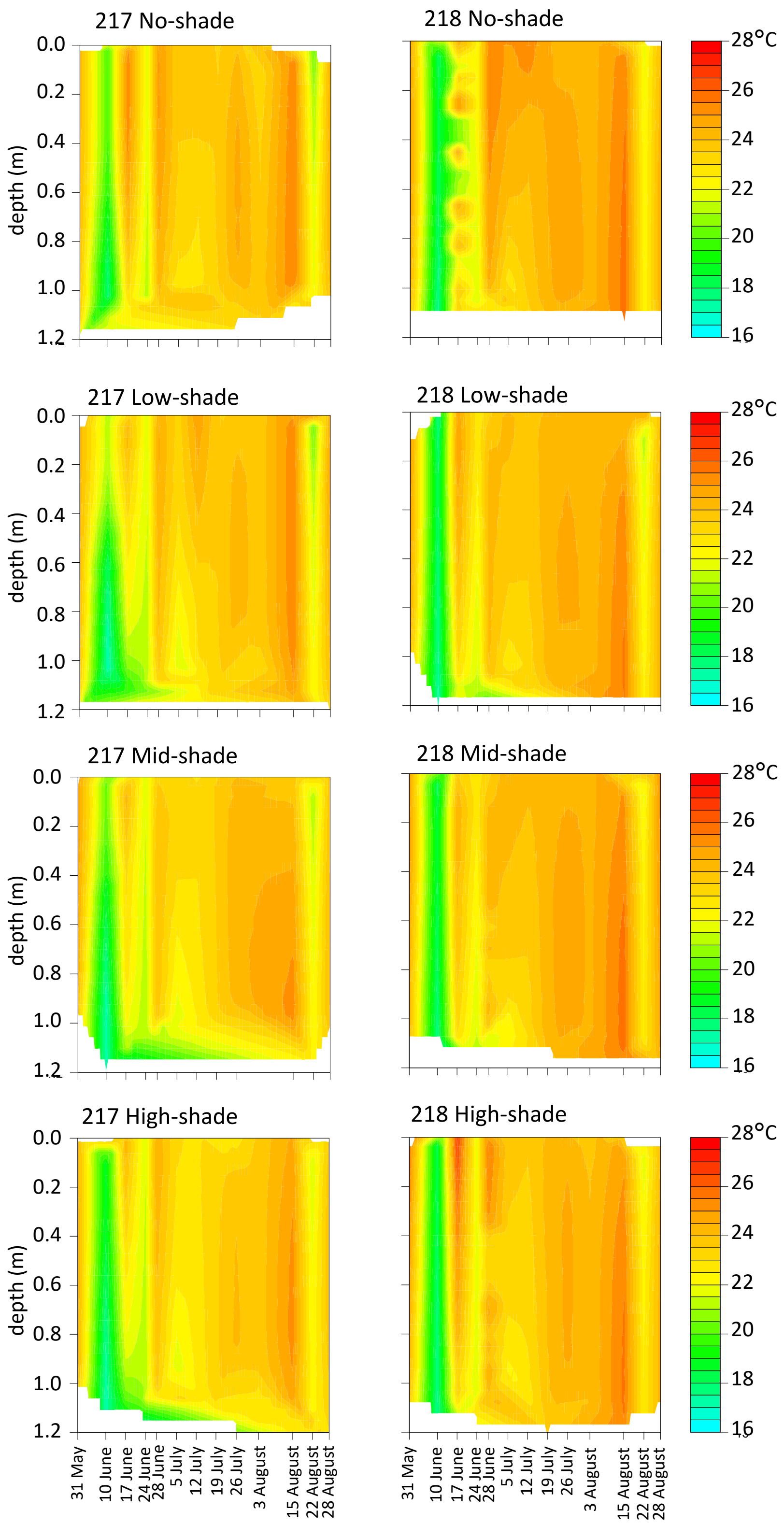

Supplementary Figure 2. Vertical profiles of water temperature in the no-shade (upper), low-shade (mid-upper), mid-shade (mid-lower), and high-shade sections (lower) of pond 217 (left) and pond 218 (right).

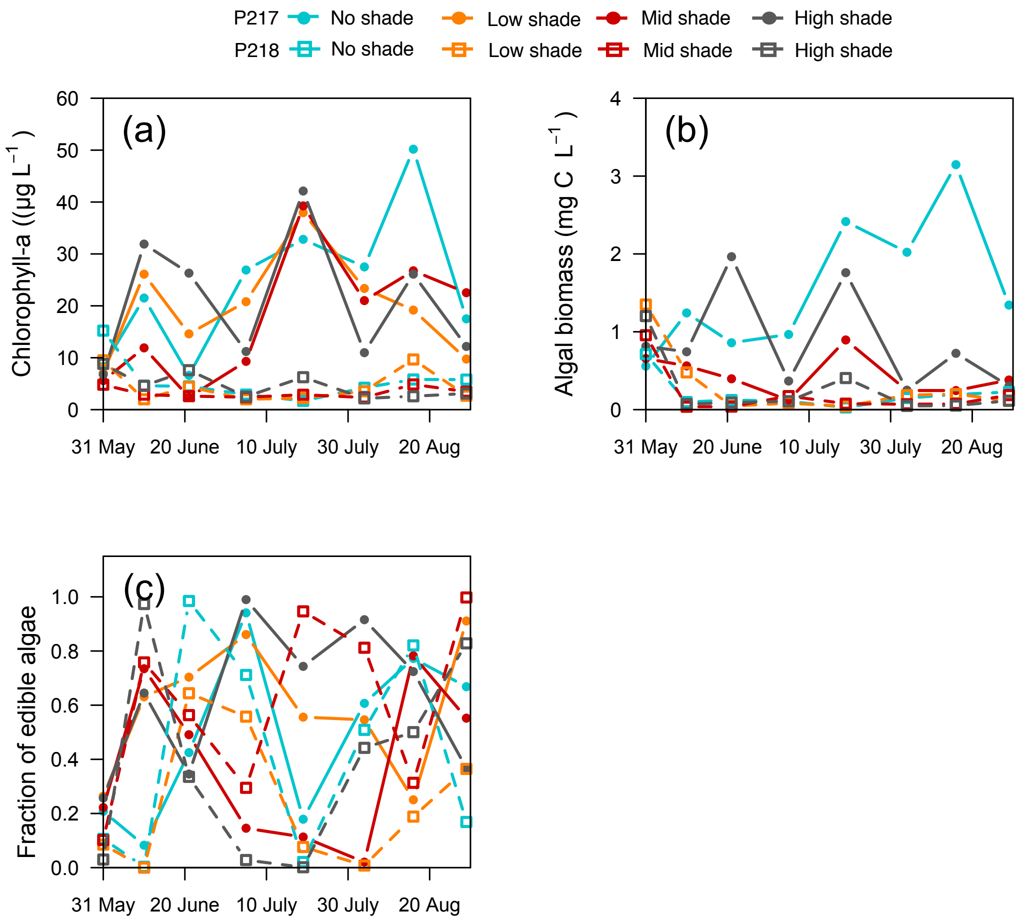

Supplementary Figure 3. Temporal changes in chlorophyll *a* (a), phytoplankton biomass (b), and fraction of edible phytoplankton (c) in the water column of no-shade (blue), low-shade (orange), mid-shade (red), and high-shade sections (gray) of pond 217 (circle and sold line) and pond 218 (square and dashed line).

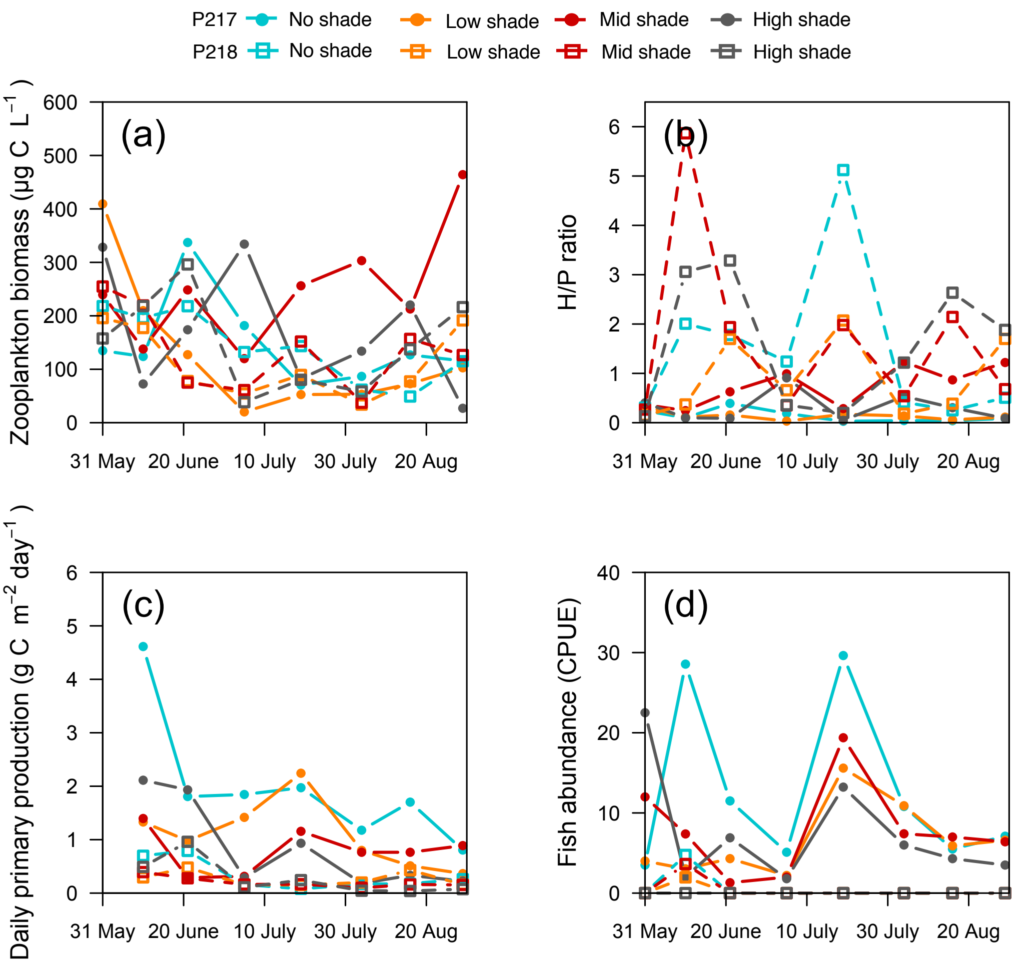

Supplementary Figure 4. Temporal changes in zooplankton biomass (a), *H*/*P* biomass ratio (b) daily production rate (c), and fish abundance (d) in the water column of no-shade (blue), low-shade (orange), mid-shade (red), and high-shade sections (gray) of pond 217 (circle and sold line) and pond 218 (square and dashed line).

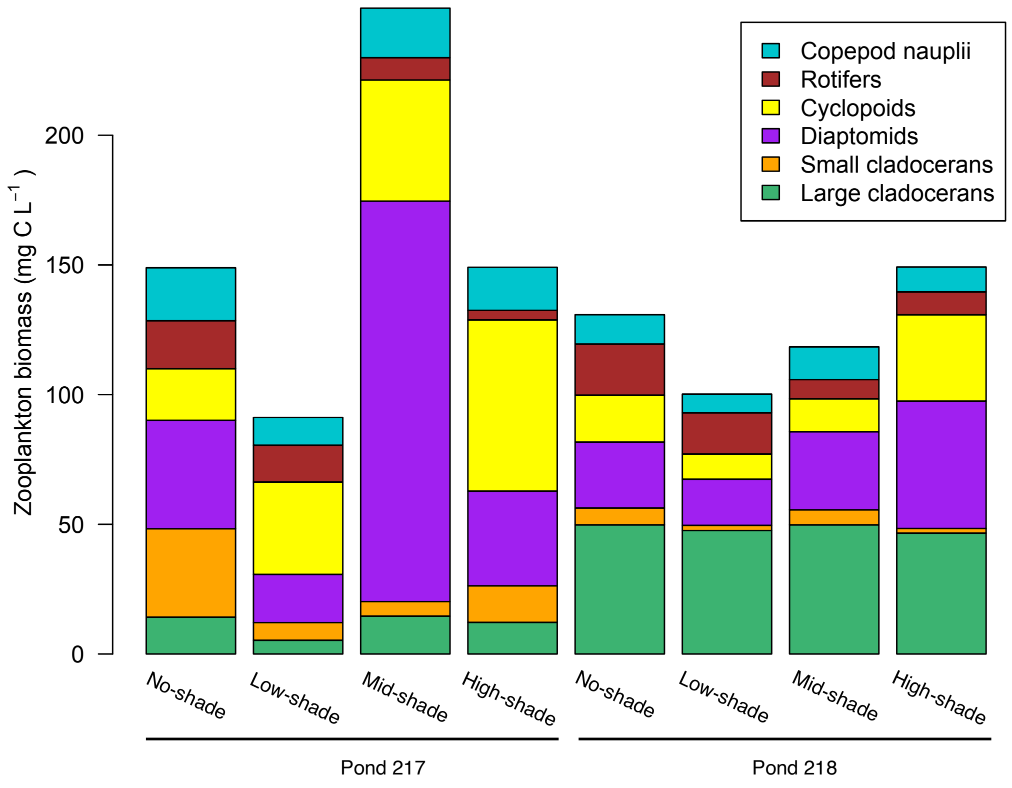

Supplementary Figure 5. Mean biomasses of zooplankton taxa during the experimental run in the no-shade, low-shade, mid-shade, and high-shade sections of pond 217 and pond 218.

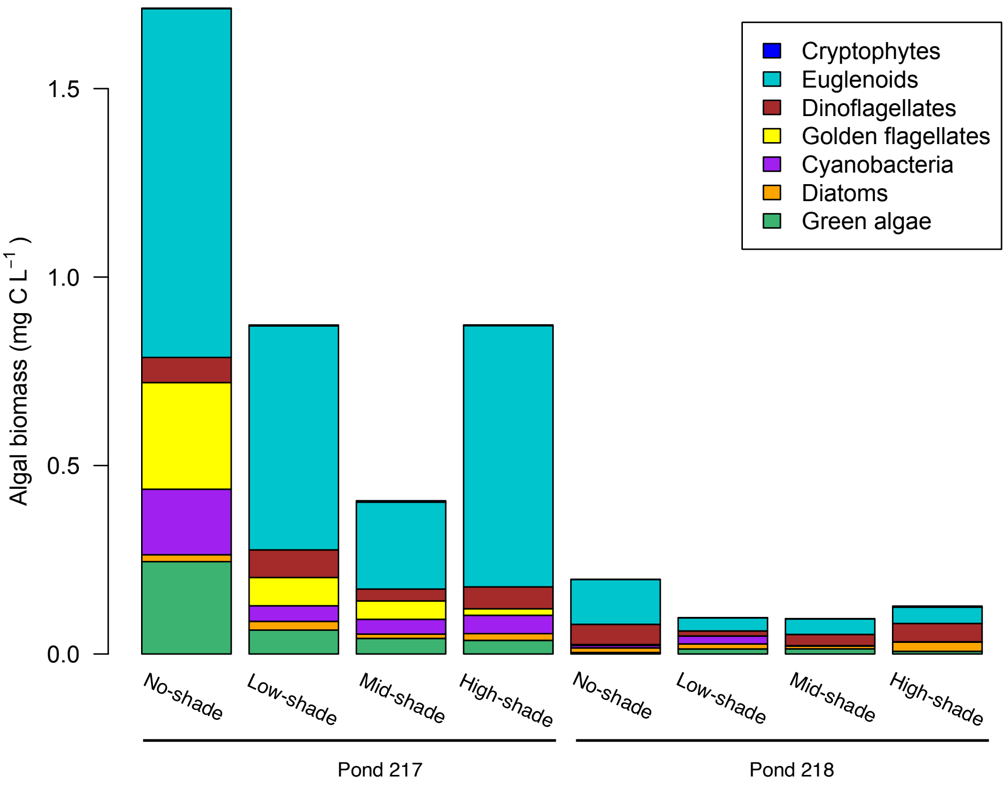

Supplementary Figure 6. Mean biomasses of phytoplankton taxa during the experimental run in the no-shade, low-shade, mid-shade, and high-shade sections of pond 217 and pond 218.

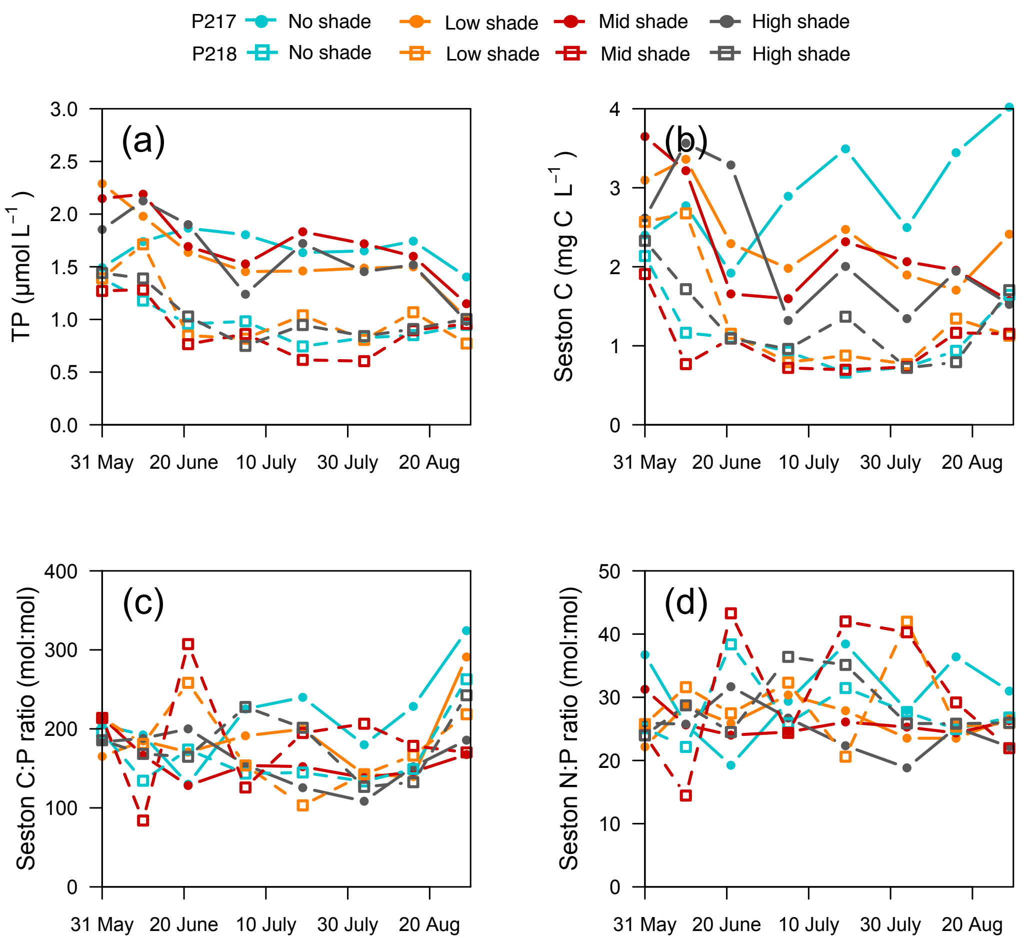

Supplementary Figure 7. Temporal changes in total phosphorus concentration (a), seston carbon (b) seston C:P ratio (c) and seston N:P ratio (d) in the water column of no-shade (blue), low-shade (orange), mid-shade (red), and high-shade sections (gray) of pond 217 (circle and sold line) and pond 218 (square and dashed line).

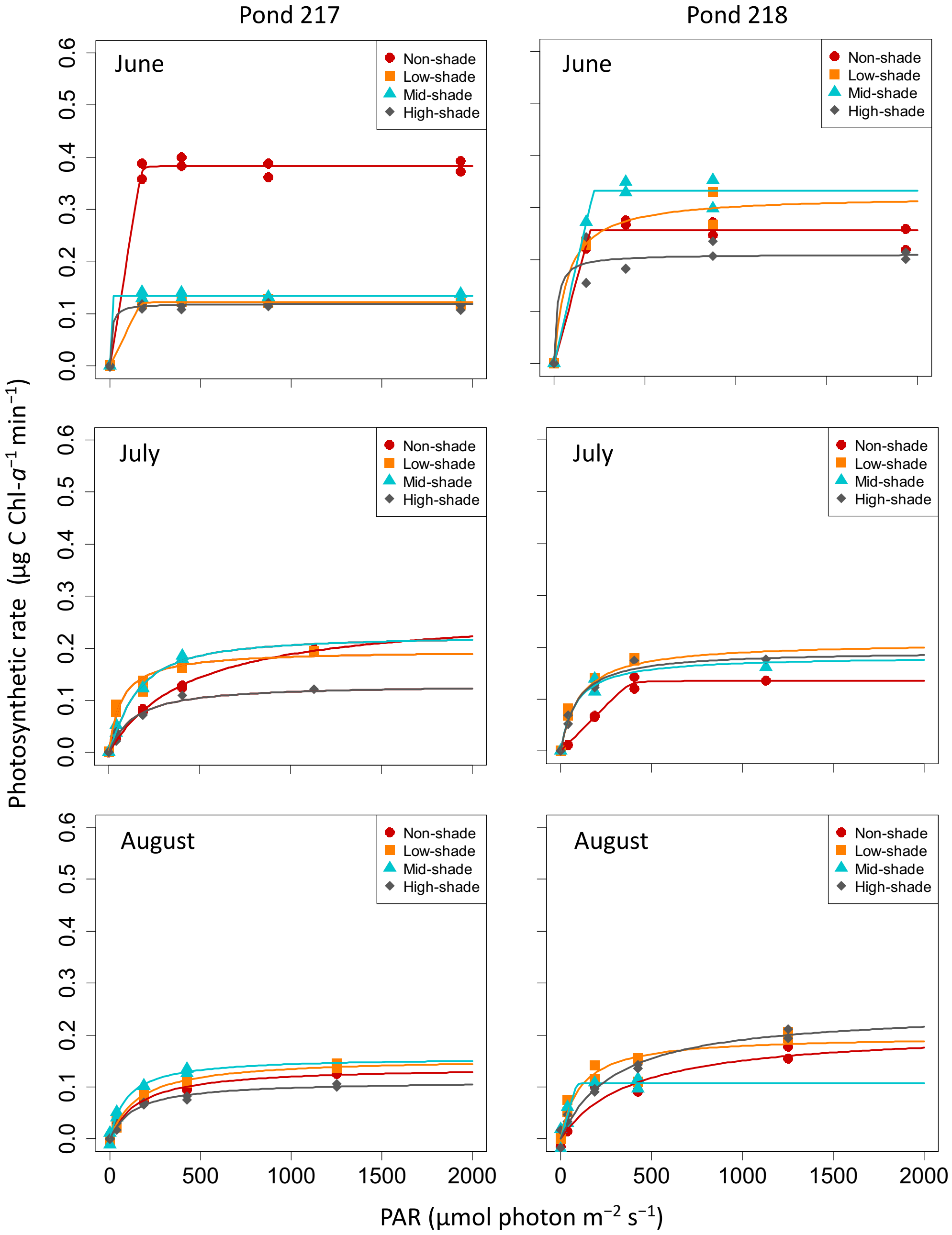

Supplementary Figure 8. Chlorophyll-*a* specific photosynthetic rates plotted against the mean PAR in June 14 (blue), July 11 (red), and August 22 (gray) in the no-shade, low-shade, mid-shade, and high-shade sections of pond 217 (upper) and 218 (lower). Each response curve was obtained by fitting data to non-rectangular hyperbola models.

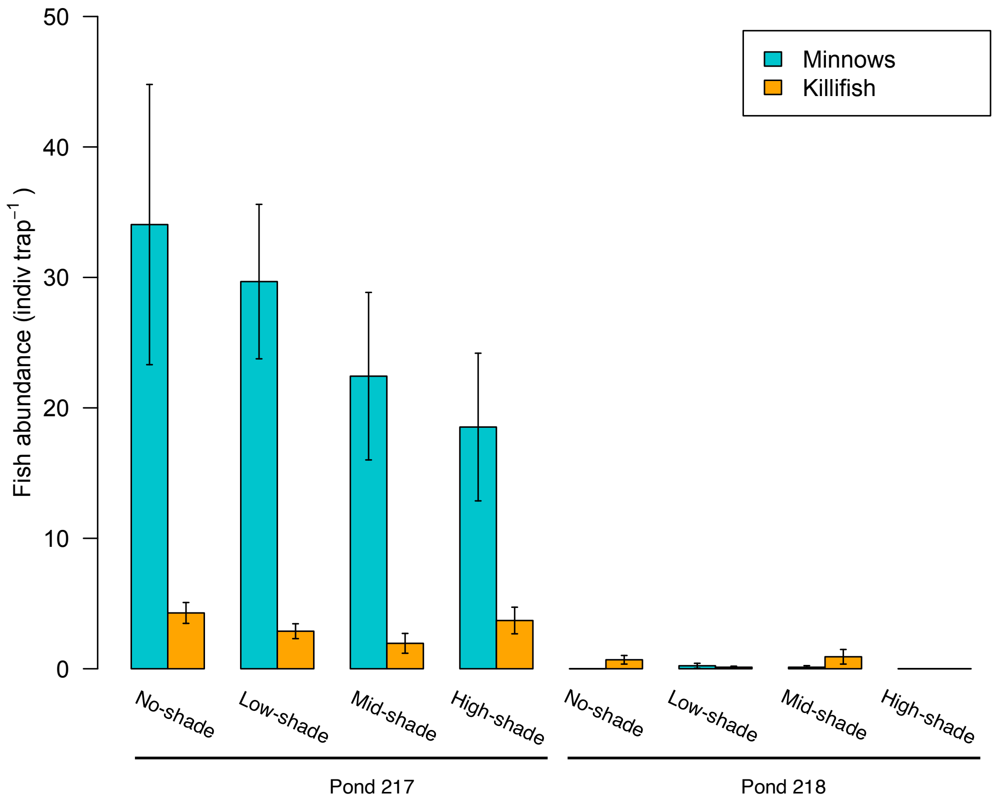

Supplementary Figure 9. Mean abundance of fish taxa during the experimental run in the no-shade, low-shade, mid-shade, and high-shade sections of pond 217 and pond 218. Vertical bars denote SE on the mean (n = 7 sampling dates).

Supplementary Table 1. Model variables.

Variable	Unit	Definition	Convert equation
P	gC m^{-2}	Producer biomass	
H	gC m^{-2}	Herbivore biomass	
g *or* $f(P)$	gC gC^{-1} d^{-1}	Biomass-specific production rate	$q_3 \times \mu^{\varepsilon 3}$
x	gC gC^{-1} d^{-1}	Biomass-specific loss rate of producers other than grazing loss	
k	0 ~ 1	Efficiency of ingested producer biomass converted into herbivore biomass	$q_1 \times \alpha_{nut}{}^{\varepsilon 1}$
f *or* $f(P)$	m^2 gC^{-1} d^{-1}	Per capita grazing rate of herbivores	
m	gC gC^{-1} d^{-1}	Per capita mortality rate of herbivores	$q_4 \times \theta^{\varepsilon 4}$
β	0~1	Fraction of primary production that herbivores consumed	$q_2 \times \alpha_{edi}{}^{\varepsilon 2}$
μ	gC mg chl-a^{-1} d^{-1}	Chl-*a* specific growth rate of producers	
α_{nut}	dimension less	Carbon to phosphorus ratio of producers	
α_{edi}	0 ~ 1	A trait value of primary producers determining edibility	
θ	CPUE	Abundance of carnivores	
q_1	dimension less	Conversion factor for adjusting to biomass unit.	
q_2	0 ~ 1	A factor for converting the trait to edible efficiency	
q_3	mg chl-*a* gC^{-1}	Conversion factor for adjusting to biomass unit.	
q_4	gC $CPUE^{-1}$	Specific predation rate	
$\varepsilon 1$		Effectiveness of the carbon to phosphorus ratio	
$\varepsilon 2$		Effectiveness of the edibility	
$\varepsilon 3$		Effectiveness of the specific growth rate	
$\varepsilon 4$		Effectiveness of the carnivore abundance	
γ		$\log(q_1) + \log(q_2) + \log(q_3) - \log(q_4)$	

Supplementary Table 2. Temporal means and standard errors (parenthesis) of plankton biomass, primary production rate, fish abundance, and seston elemental ratios in each treatment of pond 217 and 218 during the experiment.

Pond	Treatment	H/P mass ratio	Zooplankton Biomass	Algal Biovolume	Algal Biomass
		gC gC^{-1}	µg C L^{-1}	x10^{6} µm^{3} mL^{-1}	mg C L^{-1}
217	No shade	0.126 (0.049)	148.9 (34.1)	14.146 (2.676)	1.713 (0.320)
217	Low shade	0.112 (0.019)	91.3 (23.8)	6.819 (1.549)	0.873 (0.200)
217	Mid shade	0.781 (0.155)	248.9 (43.5)	3.361 (0.728)	0.407 (0.097)
217	High shade	0.296 (0.122)	149.1 (39.5)	6.510 (1.961)	0.873 (0.267)
218	No shade	1.617 (0.639)	130.8 (23.8)	1.543 (0.382)	0.132 (0.024)
218	Low shade	1.009 (0.296)	100.3 (22.8)	0.891 (0.255)	0.164 (0.057)
218	Mid shade	1.927 (0.716)	118.3 (24.2)	0.716 (0.168)	0.094 (0.023)
218	High shade	1.804 (0.475)	149.3 (36.6)	0.884 (0.383)	0.127 (0.047)

Pond	Treatment	Chlorophyll-a	Fraction of edible algae	Specific Daily Production	Daily Production
		mg chl-a m^{-2}		mgC mg chl-a^{-1} d^{-1}	gC m^{-2} d^{-1}
217	No shade	25.51 (5.00)	0.525 (0.198)	108.1 (37.3)	1.989 (0.465)
217	Low shade	21.15 (3.32)	0.637 (0.241)	50.3 (6.4)	1.092 (0.242)
217	Mid shade	18.57 (4.53)	0.406 (0.153)	59.3 (16.2)	0.796 (0.153)
217	High shade	22.41 (4.43)	0.676 (0.255)	34.5 (9.8)	0.858 (0.316)
218	No shade	4.02 (0.54)	0.460 (0.174)	81.7 (23.5)	0.334 (0.108)
218	Low shade	3.62 (0.99)	0.263 (0.099)	84.2 (14.4)	0.267 (0.052)
218	Mid shade	2.93 (0.31)	0.669 (0.253)	72.9 (15.1)	0.202 (0.038)
218	High shade	3.93 (0.77)	0.444 (0.168)	56.1 (17.5)	0.280 (0.128)

Pond	Treatment	Fish abundance	Seston C	Seston C:P ratio	Seston N:P ratio
		CPUE (gWW trap^{-1})	µmol L^{-1}	mol mol^{-1}	mol mol^{-1}
217	No shade	14.03 (4.00)	250 (22)	217.14 (22.78)	29.7 (2.45)
217	Low shade	6.93 (1.80)	192 (17)	188.44 (19.21)	26.7 (0.97)
217	Mid shade	7.27 (2.24)	171 (18)	150.29 (5.40)	25.2 (0.34)
217	High shade	5.39 (1.49)	178 (29)	159.16 (12.89)	24.7 (1.55)
218	No shade	0.68 (0.68)	85 (10)	163.11 (17.37)	28.2 (2.00)
218	Low shade	0.28 (0.28)	104 (21)	174.79 (19.27)	29.3 (2.59)
218	Mid shade	0.52 (0.52)	75 (7)	181.01 (26.44)	30.8 (4.26)
218	High shade	0.00 (0.00)	99 (13)	180.53 (16.98)	28.9 (1.84)

Supplementary Table 3. Results of generalized linear models with the five lowest Akaike's Information Criterion values. Parameter meanings are shown in Table. S1.

Model	AIC
$\log(H/P) \sim \log(\alpha_{nut}) + \log(\alpha_{edi}) + \log(\mu) + \log(\theta)$	12.20
$\log(H/P) \sim \log(\alpha_{nut}) + \log(\mu) + \log(\theta)$	16.07
$\log(H/P) \sim \log(\alpha_{nut}) + \log(\alpha_{edi}) + \log(\theta)$	20.55
$\log(H/P) \sim \log(\alpha_{edi}) + \log(\mu) + \log(\theta)$	24.58
$\log(H/P) \sim \log(\alpha_{nut}) + \log(\alpha_{edi}) + \log(\mu)$	29.10
