# Supplementary material for: A unified framework for herbivore-to-producer biomass ratio reveals the relative influence of four ecological factors
Source: Commun Biol. 2021 Jan 8;4:49. doi: 10.1038/s42003-020-01587-9 (PMC7794211; doi:10.1038/s42003-020-01587-9)
Supplement: Supplementary file 3 — Reporting Summary [file 42003_2020_1587_MOESM3_ESM.pdf]

nature research

Corresponding author(s): Jotaro Urabe

Last updated by author(s): 16/11/2020

Reporting Summary

Nature Research wishes to improve the reproducibility of the work that we publish. This form provides structure for consistency and transparency in reporting. For further information on Nature Research policies, see our Editorial Policies and the Editorial Policy Checklist.

Statistics

For all statistical analyses, confirm that the following items are present in the figure legend, table legend, main text, or Methods section.

n/a	Confirmed	
☐	☒	The exact sample size (n) for each experimental group/condition, given as a discrete number and unit of measurement
☐	☒	A statement on whether measurements were taken from distinct samples or whether the same sample was measured repeatedly
☐	☒	The statistical test(s) used AND whether they are one- or two-sided<br>*Only common tests should be described solely by name; describe more complex techniques in the Methods section.*
☐	☒	A description of all covariates tested
☐	☒	A description of any assumptions or corrections, such as tests of normality and adjustment for multiple comparisons
☐	☒	A full description of the statistical parameters including central tendency (e.g. means) or other basic estimates (e.g. regression coefficient) AND variation (e.g. standard deviation) or associated estimates of uncertainty (e.g. confidence intervals)
☐	☒	For null hypothesis testing, the test statistic (e.g. F, t, r) with confidence intervals, effect sizes, degrees of freedom and P value noted<br>*Give P values as exact values whenever suitable.*
☒	☐	For Bayesian analysis, information on the choice of priors and Markov chain Monte Carlo settings
☒	☐	For hierarchical and complex designs, identification of the appropriate level for tests and full reporting of outcomes
☐	☒	Estimates of effect sizes (e.g. Cohen's d, Pearson's r), indicating how they were calculated

Our web collection on statistics for biologists contains articles on many of the points above.

Software and code

Policy information about availability of computer code

Data collection	no soft ware used
Data analysis	we used R packages "vegan" and "car" with software R version 3.2.1.

For manuscripts utilizing custom algorithms or software that are central to the research but not yet described in published literature, software must be made available to editors and reviewers. We strongly encourage code deposition in a community repository (e.g. GitHub). See the Nature Research guidelines for submitting code & software for further information.

Data

Policy information about availability of data

All manuscripts must include a data availability statement. This statement should provide the following information, where applicable:

- Accession codes, unique identifiers, or web links for publicly available datasets
- A list of figures that have associated raw data
- A description of any restrictions on data availability

All the data used in this study are archived in Dryad (https://doi.org/10.5061/dryad.p8cz8w9ms) and will be disclosed after acceptance. However, For pear review, however, we disclose all the data that can be down loaded from following url,
https://datadryad.org/stash/share/DXnkxcQSGPejNuuvwkDqfcsFBuD02qc_4etyCtTHNqo

Field-specific reporting

Please select the one below that is the best fit for your research. If you are not sure, read the appropriate sections before making your selection.

☐ Life sciences ☐ Behavioural & social sciences ☒ Ecological, evolutionary & environmental sciences

For a reference copy of the document with all sections, see nature.com/documents/nr-reporting-summary-flat.pdf

Ecological, evolutionary & environmental sciences study design

All studies must disclose on these points even when the disclosure is negative.

Study description	To examine usefulness of the mathematical model we developed in this study, we made an experiment using experimental ponds where we manipulated light input and fish abundance.
Research sample	We sampled regularly phytoplankton, zooplankton and fish in the experimental ponds biweekly. Major taxa are Cryptohytes, Euglenodis, Dinoflagellates, Golden flagellates, Cyanobacteria, Datoms and Green algae in phytoplankton samples, Cyclopoid and Diaptomid copepods, Rotifers, and Cladocerans including Daphnia and Bosimina in zooplankton samples, and minnows (Pimephales promelas) and Killifish (Fundulus diaphanu) in fish samples.
Sampling strategy	We collected 11L of pond water for Phytoplankton samples and 30L for zooplankton. These water volumes were determined to estimate their abundances accurately based no researcher's experience. Plankton in these samples were fixed and used for estimating the abundance and biomass. Fish were collected by minnow traps. After estimating the wet weight, fish individuals collected were released near ponds that ware not used in the experiment.
Data collection	In each of samples, phytoplankton and zooplankton were enumerated according to taxa and species or genus by Kazama, Tokita, Yin, Doi and Urabe. Fish abundances were recorded by Kazama and Tokita.
Timing and spatial scale	For each of the pond used in the experiment, sampling was performed from 4 June to 28 August, 2016. During this period, samplings were done biweekly to estimate average abundances of phyto- and zooplankton that were used for examining usefulness of the mathematical model that this study has developed.
Data exclusions	All of phytoplankton, zooplankton and fish data were used in the study.
Reproducibility	In this study, we did not make replications for each treatment since it was difficult to set precisely abundance of fish in the pond units. Instead, we analyzed statistically if the response variables (phytoplankton and zooplankton abundances) were related significantly with manipulations. By doing so, we ascertained that our findings were not occurred by a chance.
Randomization	In this study, we prepared 4 different treatments for each of the two ponds. In each ponds, we assigned these treatments randomly into four sections of each pond. The random assignment was done using a hand-maid lottery by Kazama, Yamamichi and Tokita.
Blinding	Data collections and data analysis were made by different persons in the research groups at first. In plankton enumeration, we randomely assigned samples for different parsons who undertook enumeration of plankton cells and individuals as if cutting playing cards. By doing so, we avoided that data used in the analysis were affected by any of researcher's intentions.

Did the study involve field work? ☒ Yes ☐ No

Field work, collection and transport

Field conditions	The study was done from late spring to mid summer.
Location	The field experiments was performed in the Cornell University Experimental Ponds Facility (CUEPF) in Ithaca, NY, USA (42°30'N, 76° 26'W).
Access & import/export	We accessed CUEPF by cars using public loads.
Disturbance	Since these are artificial ponds, we did not make any natural disturbance by this study.

Reporting for specific materials, systems and methods

We require information from authors about some types of materials, experimental systems and methods used in many studies. Here, indicate whether each material, system or method listed is relevant to your study. If you are not sure if a list item applies to your research, read the appropriate section before selecting a response.

Materials & experimental systems

n/a	Involved in the study
☒	☐ Antibodies
☒	☐ Eukaryotic cell lines
☒	☐ Palaeontology and archaeology
☐	☒ Animals and other organisms
☒	☐ Human research participants
☒	☐ Clinical data
☒	☐ Dual use research of concern

Methods

n/a	Involved in the study
☒	☐ ChIP-seq
☒	☐ Flow cytometry
☒	☐ MRI-based neuroimaging

Animals and other organisms

Policy information about studies involving animals; ARRIVE guidelines recommended for reporting animal research

Laboratory animals	Non
Wild animals	Zooplankton individuals in 30L of the pond water collected at each sampling were fixed by methanol and all of the individuals were used to enumeration for estimating the biomass. Fish individuals collected by minnow traps were identified, measured in there wet weights and released into other ponds in the the Cornell University Experimental Ponds Facility (CUEPF).
Field-collected samples	As above, we measured wet weight biomass of fish at shores of the ponds. Zooplankton samples were processed in laboratories.
Ethics oversight	Work with fish was permitted under Cornell University's Institutional Animal Care and Use Committee protocol 2016-0095

Note that full information on the approval of the study protocol must also be provided in the manuscript.
